# Editorial: Industrial and Host Associated Stress Responses in Food Microbes. Implications for Food Technology and Food Safety

**DOI:** 10.3389/fmicb.2017.01522

**Published:** 2017-08-08

**Authors:** Lorena Ruiz, Abram Aertsen, Christophe Nguyen-The, Michael G. Gänzle, Avelino Alvarez-Ordóñez

**Affiliations:** ^1^Department of Nutrition, Food Science and Food Technology, Complutense University of Madrid Madrid, Spain; ^2^Department of Microbiology and Biochemistry of Dairy Products, Instituto de Productos Lácteos de Asturias-CSIC Villaviciosa, Spain; ^3^Laboratory of Food Microbiology, Department of Microbial and Molecular Systems, KU Leuven Leuven, Belgium; ^4^Institut National de la Recherche Agronomique, UMR408 Sécurité et Qualité des Produits d'Origine Végétale Avignon, France; ^5^Univ-Avignon, UMR408 Sécurité et Qualité des Produits d'Origine Végétale Avignon, France; ^6^Department of Agricultural, University of Alberta, Food and Nutritional Science Edmonton, AB, Canada; ^7^Department of Food Hygiene and Technology and Institute of Food Science and Technology, University of León León, Spain

**Keywords:** food microbiology, spoilage bacteria, pathogens, food preservation, bacterial stress response, stress response, food safety, food quality

Throughout the food chain, from primary production to consumption, food associated bacteria are confronted with a range of intrinsic and extrinsic factors which influence bacterial survival and activity. Intrinsic stress factors are imposed by food characteristics like high osmolarity or acidity or the dynamics of the microbial ecology of foods; and extrinsic stress factors are inflicted by food preservation methodologies, such as thermal and/or non-thermal inactivation treatments, refrigeration temperatures, or freeze-drying and host associated factors like the stomach acidity or the presence of bile salts in the intestine (Papadimitriou et al., 2016). The accompanying plethora of stress responses elicited by food associated microorganisms has important implications for food technology, quality, and safety (Alvarez-Ordoñez et al., 2015). Resistance development of pathogenic and spoilage microorganisms may impose health risks for the consumer and impart great economic losses to food industries, whereas reduced survival of probiotic or beneficial microbes may strongly compromise their functional attributes.

In the last decades substantial research efforts in the field of food microbiology have been devoted to unravel the mechanisms underlying stress responses and resistance development in food associated microorganisms in order to better predict and improve (i) the inactivation of food-borne pathogens and spoilage microorganisms and (ii) the control of the performance of starter cultures and probiotic microorganisms. Moreover, the recent implementation of system-wide omics and (single-)cell biology approaches has greatly boosted our insights into the molecular mechanisms underlying microbial inactivation and survival (Abhyankar et al., 2017). In this context, this Research Topic aims to provide an avenue to disseminate latest results on food microorganisms stress responses and adaptation, including the implementation of new methodologies for their study.

This Research Topic comprises a collection of 24 articles, including 4 reviews, 1 mini-review, 1 hypothesis and theory article, and 18 original research articles providing a broad overview of recent advances within the field of microbial stress responses and adaptation. Contributions include research on a range of pathogenic microorganisms, such as *Escherichia coli, Listeria monocytogenes, Yersinia*, and spore formers from the genus *Bacillus*, but also on microorganisms of industrial interest as starter cultures, including strains of *Leuconostoc* and *Lactobacillus*. Heat stress, acid exposure, low temperature, and high CO_2_ pressure are among the stress factors evaluated throughout the articles presented in this Research Topic. In addition, the effect of beneficial bacteria on the inactivation of pathogenic bacteria on fermented products, the effect of fermentation associated factors in the dynamics of food microbiota, and the development of new technologies for the study of the performance and inactivation of food microorganisms have also been evaluated in articles included in this research topic as summarized below.

The review by Li and Gänzle provides a comprehensive evaluation on the heat resistance of *E. coli* cells including information on the molecular mechanisms associated to heat tolerance variability among strains and on genetic determinants of extreme heat resistance. In addition, the increased heat tolerance following pre-exposure to other stresses such as desiccation or exposure to osmotic or acid stresses, and the effect of certain food ingredients on *E. coli* heat susceptibility are also discussed. Also in relation to *E. coli* heat stress, Huertas et al. demonstrate that the response of *E. coli* to thermal treatments strongly depends on the heating rates, thus providing evidence that classical estimation of heat inactivation parameters, such as D and z values, under isothermal conditions might not provide a realistic estimation of inactivation levels achieved during heat processing of food.

Molecular mechanisms allowing *E. coli* growth under mild acidic conditions were characterized by Vivijs et al. These authors identify in their elegant study new mechanisms involved in the growth of *E. coli* at moderately low pH, and demonstrate that these mechanisms differ from those used to withstand exposure to extremely low pH. The response of food pathogens to weak acids is very relevant in the context of food safety and preservation, since weak acids are frequently employed as food additives. In fact, in another work by Lenzi et al. the effect of weak acids on Shiga toxin production by an *E. coli* strain was also studied. Results presented in their work highlight the necessity to evaluate not just the bactericidal effects of technological and preservation treatments, but also other physiological characteristics of surviving bacteria, like toxin production, which might be enhanced by certain stressors.

Food components can have a strong influence on *E. coli* physiology and persistence as demonstrated by Crozier et al. and Crozier et al. who studied the transcriptional adaptation of this microorganism to vegetable extracts and its long term persistence on different live plants. Their results identify possible markers to be used in the development of risk-based analysis on microbial contamination of crops.

Overall, the research works included in this Research Topic reflect the existence of strong intra-species variability in *E. coli* tolerance to food associated stress factors. This fact was further highlighted in the work by Elhadidy and Alvarez-Ordóñez, who evaluated the response of a collection of strains to multiple stresses prevailing throughout the food chain. These authors report that *E. coli* serotypes more commonly associated with human disease do also exhibit higher resistance to food-associated stresses, what suggests the influence that stressors may have on the spread of this human pathogen.

Bacterial stress resistance is of particular concern in those species exhibiting specialized persistence mechanisms as is the case of sporulated bacteria and/or biofilm formers. Six original articles and one review article in this Research Topic focus on sporulated *Bacillus* species and one original research article focuses on characteristics of biofilm-lifestyle cells. From these, four articles study spores inactivation dynamics through heat and high pressure CO_2_ treatments, while two articles are conducted with vegetative forms. Regarding spores, the study by Berendsen et al. identify genetic elements in several *Bacillus* species conferring high heat tolerance to the spores, thus providing new knowledge to design genetic-based detection methods to predict the heat tolerance of the spores produced. In another work, a strong variability in the recovery rate of heat treated spores depending on the media used was reported by Warda et al. This work also studies a range of deletion mutants with defects in spore recovery, providing new knowledge on the specific function of genes required for spore germination and emphasizing the importance of optimizing both preservation treatments and methodological procedures employed for their evaluation. Whereas, heat is the most commonly used method to inactivate spore formers, Rao et al. and Rao et al. also report in two research articles that high pressure CO_2_ inactivates spores by damaging the spore structure, allowing them to demonstrate the synergistic effect of high pressure CO_2_ and nisin for *B. subtilis* spores inactivation. These works highlight that an advanced understanding of the inactivation mechanisms can facilitate the rationale for selection of preservation treatments.

The stress response of vegetative forms of *Bacillus* spp. is evaluated in two original research articles. Van Beilen et al. screened for *B. cereus* mutants susceptible to weak organic acids commonly used as food preservatives. As a result, a gene essential for membrane homeostasis maintenance and sorbic acid tolerance was identified. In another work with vegetative forms of *Bacillus*, Kranzler et al. showed that heat tightly controls the production of the emetic toxin cereulide produced by *B. cereus*. The production of toxin and the proportion of toxin isomers differing in toxicity were shown to be strongly influenced by the temperature, allowing the authors to conclude that toxin production cannot be predicted from growth rates. This emphasizes the necessity to evaluate bacterial traits in preserved food and not just growth parameters or sole cell numbers.

One last article focused on *Bacillus* species is presented by Duport et al. who comprehensively review aspects of bacterial response and adaptation to gastrointestinal stress factors including acidity and fluctuating oxygen availability, and the role of these factors on enterotoxin production. They also identify knowledge gaps providing a basis to delineate future research on this topic.

Another common basis for bacterial persistence along the food chain is based on the biofilm forming capability of foodborne pathogenic and spoilage bacteria, since biofilm-living cells are more resistant to biocides and disinfection regimes. In the work by Dubois-Brisonnet et al. biofilm lifestyle in *Staphylococcus, Listeria, Pseudomonas*, and *Salmonella* species is associated with changes in membrane fatty acid composition, which are hypothesized as a biofilm-adaptive trait allowing energy saving and long-term survival. Among biofilm forming microorganisms *Listeria monocytogenes* represents a great concern to food safety due to the high mortality rate of listeriosis and, therefore, several articles in this Research Topic focus on this bacterium, which is a highly adaptable microorganism capable to survive under a range of stress factors. NicAogáin and O'Byrne review routes allowing *L. monocytogenes* inclusion in the food chain, genetic mechanisms allowing its survival under stressors encountered throughout the food chain, and describe control mechanisms, including novel inactivation approaches such as those targeting specific regulatory elements. Tremonte et al. studied in a range of foodborne pathogens, the phylogenetic conservation, and tridimensional structure prediction of a universal stress protein whose production is acid-induced in *Listeria* and has been related to acid tolerance. Another concerning feature regarding *L. monocytogenes* control is its psychrotrophic character. In this context, Saunders et al. modeled the activity of the enzyme initiating fatty acids biosynthesis at different temperatures, providing new knowledge on the basis of the homeoviscous adaptation to low temperature in *L. monocytogenes* and offering new targets to control *L. monocytogenes* survival under refrigeration temperatures.

Proteins involved in adaptation to cold shock in *Yersinia*, a psychrotrophic member of the *Enterobacteriaceae* family, capable to grow at temperatures close to 0°C, have also been reviewed in the article by Keto-Timonen et al. In other bacteria, these proteins are involved in adaptation to a range of stress factors and, therefore, studies on their regulation and specific activity in relevant food microorganisms is warranted.

Other original research articles included in this Research Topic are related to stresses imposed by or affecting beneficial food-associated bacteria. On the one hand, Liu et al. identify bioactive cyclic peptides displaying antibacterial activity against a range of multidrug resistant bacteria in fermented Kimchi. Producing strains belong to *Leuconostoc* species and offer potential to prevent food contamination with undesirable microorganisms. On the other hand, Li et al. assess the dynamic evolution of microbiota composition during the two stage fermentation process of a traditional Chinese Daqu fermented product. Multivariable analysis allowed the authors to establish a correlation between environmental variables and microbial community profiles. These kind of studies will ease the selection of appropriate environmental parameters when establishing or improving industrial fermentation procedures.

Finally, four articles in this collection report the development and/or implementation of new methodologies to study microbial stress responses and monitor the efficacy of food preservation treatments. One of the issues that may compromise the efficacy of food preservation technologies is the existence of damaged cells, incapable of growth in standard laboratory media, but still alive and capable to proliferate in foods under appropriate conditions. In this context Espina et al. studied the selective media methodology used to determine the proportion of sublethally injured cells, using heat treated *E. coli* as a model. These authors provide a mechanistic explanation for the methodology and propose method improvements.

Similarly, Hazeleger et al. report the optimization of protocols for the detection of *Campylobacter* in foods. *Campylobacter* grow slowly during the recommended two-step enrichment process, and, therefore, can be easily outgrown by other bacteria present in the sample. In this work, authors evaluate some modifications of the recommended procedure that result in improved detection of *Campylobacter* specifically in samples where the presence of background flora is expected.

New applications of already existing methodologies are also described in this collection of articles. Bancalari et al. test a new potential application of impedance microbiology based methods to ease the evaluation in real time of acidifying performance in a large number of lactic acid bacteria. Finally, a review article by Léonard et al. discusses the potential, advantages and limitations of multi-parameter flow cytometry assays as a tool to study antimicrobial treatment efficacy. These authors also describe the cautions that must be taken when quantifying both inactive and damaged cells and discuss additional technologies that may be used to corroborate results obtained through flow cytometry based approaches.

This editorial summarizes the publications included in this Research Topic. We sincerely hope that this collection of articles will prompt further research on microbial stress responses and contribute to advance the knowledge on microbial physiology and ecology in foods.

## Author contributions

LR designed and wrote the Editorial with contributions from AA, CN, MG, and AA.

### Conflict of interest statement

The authors declare that the research was conducted in the absence of any commercial or financial relationships that could be construed as a potential conflict of interest.
